# Exploring the effectiveness of auditory, visual, and audio-visual sensory cues in a multiple object tracking environment

**DOI:** 10.3758/s13414-022-02492-5

**Published:** 2022-05-24

**Authors:** Julia Föcker, Polly Atkins, Foivos-Christos Vantzos, Maximilian Wilhelm, Thomas Schenk, Hauke S. Meyerhoff

**Affiliations:** 1 School of Psychology, College of Social Science, University of Lincoln, Lincoln, UK; 2 Center for Psychotherapy Research, University Hospital Heidelberg, Heidelberg, Germany; 3 Ludwig-Maximilians-University Munich, Munich, Germany; 4 University of Erfurt, Erfurt, Germany; 5 Leibniz-Institut für Wissensmedien, Tübingen, Germany

**Keywords:** Multisensory processing, Attention: object-based, Visual perception

## Abstract

Maintaining object correspondence among multiple moving objects is an essential task of the perceptual system in many everyday life activities. A substantial body of research has confirmed that observers are able to track multiple target objects amongst identical distractors based only on their spatiotemporal information. However, naturalistic tasks typically involve the integration of information from more than one modality, and there is limited research investigating whether auditory and audio-visual cues improve tracking. In two experiments, we asked participants to track either five target objects or three versus five target objects amongst similarly indistinguishable distractor objects for 14 s. During the tracking interval, the target objects bounced occasionally against the boundary of a centralised orange circle. A visual cue, an auditory cue, neither or both coincided with these collisions. Following the motion interval, the participants were asked to indicate all target objects. Across both experiments and both set sizes, our results indicated that visual and auditory cues increased tracking accuracy although visual cues were more effective than auditory cues. Audio-visual cues, however, did not increase tracking performance beyond the level of purely visual cues for both high and low load conditions. We discuss the theoretical implications of our findings for multiple object tracking as well as for the principles of multisensory integration.

## Introduction

The navigation through a dynamic multisensory environment requires an individual to efficiently extract and integrate the information from multiple moving objects and to combine these signals with different sensory information. A challenging task in this scenario involves tracking a set of moving objects amongst irrelevant distractors also known as multiple object tracking (MOT; Pylyshyn & Storm, 1988; see Meyerhoff et al., 2017, for a review). A MOT trial typically starts with a cueing phase that highlights the subset of objects that need to be tracked (e.g., by brief colour cues). Following this phase, the target and distractor objects remain indistinguishable across an interval of motion during which they can be tracked only based on their spatio-temporal information. At the end of each trial, the participants are asked to indicate the target objects, and tracking accuracy typically serves as a dependent variable. Across the last three decades, numerous MOT studies have probed the effects of object speed (Alvarez & Franconeri, 2007; Vul et al., 2009), set size (Alvarez & Franconeri, 2007; Bettencourt & Somers, 2009; Drew et al., 2011; Pylyshyn & Storm, 1988), tracking duration (Oksama & Hyönä, 2004; Wolfe et al., 2007), the spatial proximity between targets and distractors (Alvarez & Franconeri, 2007; Bettencourt & Somers, 2009; Franconeri et al., 2010; O’Hearn et al., 2005), motion information (Fencsik et al., 2007; Howard et al., 2011; Iordanescu et al., 2009; Meyerhoff et al., 2013; St. Clair et al., 2010), or multitasking (Allen et al., 2006; Huff et al., 2012; Tombu & Seiffert, 2008) on tracking performance. However, to our knowledge, previous studies on MOT have not addressed the question whether auditory as well as audio-visual cues are able to improve tracking performance. This question is of relevance, as real-world scenarios that might serve for the application of basic MOT typically would involve not only vision, but also other modalities such as audition. For instance, whilst driving, auditory information such as the sudden onset of an acceleration sound might influence the distribution of attention amongst the multiple “to-be-tracked” vehicles on the road. Furthermore, air traffic control involves components of MOT (e.g., Hope et al., 2010). Here, additional auditory cues might allow the operator to detect potential collisions or abrupt direction changes of an aircraft. In the present research project, we therefore aim at providing the first evidence of how auditory cues might affect tracking performance, which might provide the basis for more complete models of MOT.

### The impact of auditory cues on visual perception

Previous studies have explored the interaction between the auditory and the visual modality in different experimental scenarios, such as visual search (Gao et al., 2021; Lunn et al., 2019; Matusz & Eimer, 2011; Matusz et al., 2015; Turoman et al., 2021;Van der Burg et al., 2008), priming (Föcker et al., 2011; Hölig et al., 2017; Schneider et al., 2008), exogenous attention (Hillyard et al., 2016; Keefe et al., 2021; McDonald, 2000; Störmer et al., 2009, Störmer, 2019), or response competition (Lunn et al., 2019). Further, their application to ‘real-world scenarios’ (see Soto-Faraco et al., 2019, for an overview) as well as their development (Matusz et al., 2019) have been the subject of investigation.

Of note, auditory cues have been discussed to evoke changes in visual perception, exemplified in the sound-flash illusion in which participants perceive several illusory visual flashes when one visual flash is accompanied by multiple sounds (Shams et al., 2000). Other studies have shown that the detection of visual blinks can be improved by auditory cues (Noesselt et al., 2008) and that attentional processes such as visual search could benefit from synchronously presented sounds (pip-and-pop effect; Gao et al., 2021; Van der Burg et al., 2008). Correspondingly, Vroomen and De Gelder (2000) indicated that tones that coincide with the visual target in a stream of displays “enhance the visibility of the target” (Vroomen & De Gelder, 2000, p. 1585). Some authors have argued that the simultaneous presentation of a visual and auditory stimulus acts as a supramodal binding feature and enables the integration of the auditory and the visual signal by evoking the perception of a salient visual stimulus that “automatically attracts attention in a bottom-up fashion” (Talsma et al., 2010, p. 401;Van der Burg et al., 2008). However, a recent study has called the explanation involving multisensory integration into question and suggests that the pip-and-pop effect represents an ‘oddball’ effect instead (Gao et al., 2021). It has been argued that the simultaneous presentation of the sound in a visual search experiment changes the target into a rare ‘oddball’ stimulus, which can be easier distinguished from the more frequently presented distractors (for a similar discussion, see also Ngo & Spence, 2012; Vroomen & De Gelder, 2000). Another explanation for the pip-and-pop effect has been summarised as a ‘freezing effect’: the visual target subjectively seems to persist longer when a sound is presented simultaneously with a visual target, which is supported by longer fixation duration in the sound compared to the no-sound condition (Zou et al., 2012). Irrespective of the exact explanation, direction changes of moving objects that coincide with brief tones are more likely to be detected (Staufenbiel et al., 2011), and reveal perceptual consequences that are comparable to a direct guidance of visual attention (Meyerhoff et al., in press).

To summarise, regarding the present experiments, this line of research has shown that task-irrelevant auditory cues are able to modify visual perception and attentional processing.

### The underlying principles of audio-visual integration

Different mechanisms have been identified to explain audio-visual integration at the neural and behavioural level, by investigating the cellular response pattern of specific types of neurons, located in the superior colliculus (Stein & Stanford, 2008). These neural patterns demonstrate that the closer temporal and spatial proximity of two or more different sensory cues enhance the neural response. Conversely, cues that are presented with spatial or temporal disparity can elicit “response depression” (Stein & Stanford, 2008, p. 257).

Besides the temporal and spatially synchronous presentation of auditory and visual information, temporal regularities between auditory and visual signals could be used to anticipate specific sensory input at specific time points (Ten Oever et al., 2014). For instance, a regularly presented auditory cue can be used to temporally prepare for the occurrence of a subsequent target event (Los & Van der Burg, 2013), which also enhances the process of multisensory integration (see also Soto-Faraco et al., 2019, for a review). Other features such as semantic congruency of sensory information have been also suggested to facilitate multisensory integration (Spence, 2007).

Of note, the salience of auditory and visual information also influences the integration of different modalities. For instance, the rule of ‘inverse effectiveness’ states that “multisensory enhancement is typically inversely related to the effectiveness of the individual cues that are being combined” (Stein & Stanford, 2008, p. 257). According to this principle, unimodal cues that are already highly effective will not exceed this efficiency when combined with cues from different modalities. However, less effective unimodal cues would substantially benefit from the integration process. With regard to the present experiment, the visual signals (such as direction changes of tracked objects) within a loaded MOT display might be relatively ineffective in guiding attention, and thus are a good candidate to benefit from audio-visual integration.

### The impact of cognitive and perceptual load on audio-visual integration

One intriguing question is whether audio-visual integration requires attention and how perceptual load modulates audio-visual integration. Whereas some of the studies outlined above indicated that multisensory integration is an automatic mechanism or equivalent to a bottom-up process (Bertelson et al., 2000; Driver, 1996; Matusz & Eimer, 2011; van der Burg et al., 2008), other authors documented that attentional selection is required prior to multisensory integration, also summarised as top-down attention (Alsius et al., 2005; Talsma & Woldorff, 2005). Many different factors might explain the top-down/bottom up-debate, such as the properties of the multisensory stimuli (auditory cues vs. complex information), “salience of the material, task relevance, the experimental design, and perceptual load” (Soto-Faraco et al., 2019, p. 8).

With regards to load dependency, several studies documented that the detection of task-relevant multisensory stimuli is enhanced compared to unisensory information irrespective of the load condition (Lunn et al., 2019; Santangelo & Spence, 2007). For instance, Lunn et al. (2019) asked participants to perform a rapid serial visual search task under high load or low load while participants were asked to detect peripheral multisensory or unisensory targets. Participants were faster and more accurate in the multisensory compared to the unisensory condition, irrespective of load. However, when the participants were instructed to ignore multisensory distractors, the multisensory distractors did not elicit a stronger task interference compared to unisensory distractors, irrespective of the load condition. From these results, Lunn et al. (2019) concluded that the impact of multisensory stimuli might only unfold in those situations in which the participants are already “looking out for” an object (Lunn et al., 2019, p. 48; see Matusz et al. (2015, 2019) for the impact of perceptual load on distractor processing in children and adults).

Applying this conclusion to real-world scenarios such as driving or concentrating in a lecture would imply that multisensory distractors do not have a higher impact than unisensory distractors. By contrast, sensory cues to which a driver is already attending might be more impactful when presented under multisensory compared to unisensory conditions.

### Visual cues improve multiple object tracking (MOT) performance

Even though previous literature has not considered auditory cues during multiple object tracking, the addition of visual features to the moving objects has been shown to improve tracking performance (Bae & Flombaum, 2012; Cohen et al., 2011; Drew et al., 2009, 2013; Horowitz et al., 2007; Howe & Holcombe, 2012; Liu & Chen, 2012; Makovski & Jiang, 2009a, 2009b; Papenmeier et al., 2014; Pylyshyn, 2006; Ren et al., 2009). For instance, Bae and Flombaum (2012) showed that brief colour changes of the distractor objects during moments of spatial proximity with the targets improved MOT performance. This finding suggests that the participants were able to use the colour information to maintain the target objects during those moments at which confusion errors are the most likely (Drew et al., 2013). On the theoretical level, an interesting line of research has argued that tracking is based on a target recovery process. According to this model, target information can be updated based on the colour information that is stored in visual working memory (Makovski & Jiang, 2009b). In line with this account, Papenmeier et al. (2014) showed that if the spatio-temporal information during tracking decreased in reliability, object colour is used reflexively to infer the spatial locations of the tracked objects. In this study, maintaining object colours across spatio-temporal discontinuities preserved tracking performance, whereas changing the colour information from the target to the distractor at the moment of discontinuity impaired tracking performance. This pattern follows a more general flexible-weighting view (Hein & Moore, 2012), according to which spatiotemporal information and surface features are both used to establish object correspondence and weighted according to the reliability of the available information.

In sum, this research demonstrated that visual cues that allow for a direct or indirect (re)identification of the target object during tracking can be successfully picked up in order to improve tracking performance. In the present project, we aimed to extend these findings to audio-visual cues. Therefore, we examined whether auditory and audio-visual cues can improve tracking performance, and how they add to or interact with visual cues.

Our main aim in the current set of experiments was to investigate whether target enhancement occurs for auditory or audio-visual cues, and how they relate to purely visual cues. Derived from previous MOT research, we expected that visual cues will improve tracking performance relative to a no-cue condition.

### Experimental design

With regards to this question, we considered two findings from research on audio-visual attention to be of particular interest. The first line of research addresses the impact of auditory information on visual attention. Based on the research outlined above, we developed a variant of the MOT paradigm in which we presented brief tones simultaneously to the visual bouncing events of target objects in order to turn this object to an “oddball event” that captures the participant’s attention. Across all targets, the bouncing events occurred in regular time intervals, which allows the participant to create predictions about upcoming sensory cues.

The participants in our experimental design were instructed in advance that only the target objects bounced against the inner orange circle and that this would coincide with a sensory cue. Further, we used a blocked design in which the different conditions were presented subsequently (rather than mixed on a trial-by-trial basis), to maximise the use of the different sensory cues (see corresponding procedure in Blau et al., 2009; Guerreiro et al., 2015; Hein et al., 2007; Robins et al., 2009; van Atteveldt et al., 2004; van der Burg et al., 2013). Our paradigm therefore addresses top-down rather than bottom-up processing.

The second line of research on audio-visual interactions that we considered to be relevant for MOT addresses the question of whether (and how) coinciding tones alter perceived object correspondence. For instance, there are numerous studies demonstrating that a brief tone affects how the visual system resolves the motion paths of dynamic objects (bouncing vs. streaming; Grassi & Casco, 2009; Meyerhoff & Suzuki, 2018; Sekuler et al., 1997). Comparable effects also emerge when visual cues alone such as a flash are presented instead of tones (Adams & Grove, 2018; Burns & Zanker, 2000; Kawabe & Miura, 2006; Watanabe & Shimojo, 1998, 2001).

**Fig. 1:**
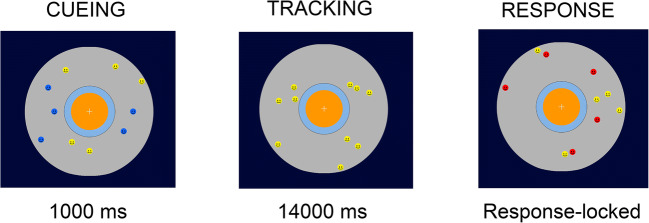
Multiple object tracking task. A trial started with the movement of blue objects (targets) and yellow objects (distractors) for a duration of 1000 ms. After 1000 ms, all objects turned yellow (tracking phase, duration: 14000 ms). During the tracking interval, targets bounced occasionally against the inner orange central and elicited a visual cue, an auditory cue, both, or no cue. The movement of the objects stopped after 14000 ms and participants had to select the target objects (mark all procedure)

Together, both of these lines of research build a foundation that suggests that auditory cues might influence tracking due to the cues guiding visual attention towards the tracked objects, and may help to (re)locate the target or to establish object correspondence. To investigate this hypothesis, we designed two experiments in which we manipulated the presence of auditory and visual cues when the target objects bounced off an inner circle of the tracking area. In both experiments, the participants tracked five target objects among five additional distractor objects. During the tracking interval, an auditory, visual, audio-visual or no cue was presented when one of the targets bounced against an inner boundary of the tracking area. Following the tracking interval, the participants were asked to select the target objects via mouse click after the movement of the objects stopped (see Fig. 1).

We chose such direction changes as they reflect a visual transient of which tone might increase the salience. Further, a direction change is a critical moment during tracking as it increases the difficulty in establishing object correspondence (Meyerhoff et al., 2013).

In the second experiment, we additionally manipulated tracking load (five targets in Experiment 1; three vs. five targets in Experiment 2) in order to understand the impact of audio-visual cues under different load conditions (see also Alsius et al., 2005; Santangelo & Spence, 2007).

With regards to the effectiveness of the audio-visual cues, there are two possible outcomes: according to the multisensory enhancement account, audio-visual cues are the most salient stimuli, and it might be argued that audio-visual cues would increase tracking performance even further compared to the corresponding unisensory visual and auditory cues (Stein & Stanford, 2008). Thus, we would expect that performance in the audio-visual condition is higher compared to the unisensory visual and the unisensory auditory condition. However, according to the rule of inverse effectiveness, it might be argued that visual cues are already effective cues. Therefore, an audio-visual cue might not increase tracking performance compared to the visual cues (Stein & Stanford, 2008) as the integration would not further increase the effectiveness.

We hypothesized that auditory cues during tracking might be effective in (re)guiding visual attention toward tracked targets if they coincide with another visual transient of the target such as a direction change, which might go unnoticed in a purely visual condition.

To anticipate our main result, we observed that tracking performance improved when visual and/or auditory cues were presented, while audio-visual cues did not elicit better performance compared to purely visual cues.

## Experiment 1

### Method

We built up upon the MOT experiment reported in Dye and Bavelier (2010) and in Green and Bavelier (2006). However, besides using similar stimulus material of blue and yellow smiley faces and a circular arrangement in which the objects are located (Dye and Bavelier, 2010), we created a fully independent experiment, which we describe in the following.

#### Participants

Seventy-three participants (age range: 19–38 years, *M*: 20 years, *SD*: 2.5; 40 females) took part in Experiment 1. The sample size emerged from the following considerations: A power analysis revealed a recommended sample size of at least N = 54 to observe an effect of *f = .25* at α = 0.05 and 1-β = 0.95, in our repeated-measures ANOVA design (g*power; Faul et al., 2007, 2009) assuming a correlation between the groups of *r* = .5. As we are not aware of any other study that tested cross-modal effects in MOT, we increased this sample size in order to compensate for potentially weaker manifestations of the effect.

All participants were recruited at the University of Lincoln. Five participants were excluded from the data analysis due to outliers (see *Data analysis* section for outlier removal procedure). The final sample consisted of 68 participants (age range: 19–38 years, *M*: 20 years, *SD*: 2.6; 37 females). All experimental procedures adhered to the Declaration of Helsinki. Prior to participating in the experiment, written informed consent was obtained from all observers and the study was approved by the University of Lincoln’s Ethics Committee.

#### Apparatus and stimuli

The MOT task was designed in Unity (Version 2019.11f1).

#### Design and procedure

At the beginning of a trial, five blue target objects and five yellow distractor objects started moving on the screen for 1,000 ms (Cueing interval, see Fig. 1). Participants were asked to track the blue target objects whilst ignoring the yellow distractor objects. After 1000 ms, the blue objects turned yellow (tracking interval), so that targets and distractor objects were visually indistinguishable from each other. In the tracking interval (duration 14000 ms), the bouncing of the target against the inner orange circle was associated with a visual cue, an auditory cue, an audio-visual cue or no cue (see Fig. 1).

After the objects had stopped moving, participants were instructed to select the five objects that they believed to be the targets with the computer mouse. Participants were instructed to guess when uncertain. The objects that were indicated turned red. Participants could also correct their choice by unmarking the selected target object which then turned yellow again. Participants received feedback regarding their accuracy at the end of each trial.

The experiment was divided into eight blocks, the no-cue, the auditory cue (A), visual cue (V), and the audio-visual cue condition (AV). Each block consisted of one of these conditions and was repeated twice, thus resulting in eight blocks. The first four blocks included each condition once, which then were repeated in the remaining four blocks. The participants completed two practice trials at the beginning each block 1–4 (i.e., once for each condition). Counterbalanced across participants, these blocks were presented in four different orders: (1) AV, V, NC A, AV, V, NC, A; (2) NC, A, V, AV, NC, A, V, AV; (3) V, AV, A, NC, V, AV, A, NC; and (4) A, NC, AV, V, A, NC, AV, V.

We chose this blocked design as we were interested in the potential of different cues that might contribute to tracking. We therefore blocked our design in order to maximise the impact of the cues. This is a common procedure to elicit target enhancement and avoid any distractor effects (for a corresponding procedure, see Blau et al., 2009; Guerreiro et al., 2015; Hein et al., 2007; Robins et al., 2009; van Atteveldt et al., 2004; van der Burg et al., 2013).

The experiment consisted of 56 trials in total, 12 trials presented in each condition (no cue, audio-visual, auditory, visual condition, 4 × 12 = 48 trials), and two training trials in each condition (eight trials in total). Each block consisted of six experimental trials.

#### Stimulus material

The stimulus materials were presented with a HP Elite Display E240 computer monitor (screen size: width: 52.7 cm, height: 29.64 cm, 1,920 × 1,080 resolution, 60-Hz refresh rate). Participants were asked to position their head on a chin rest placed 60 cm away from the screen, which maintained a constant distance between the participant’s eyes and the screen. The brightness of the screen was set to 75% contrast.

Blue and yellow smiley faces (diameter: 1 cm; ~0.95°) moved within a grey circle (diameter: 20 cm, 18.9°). An orange circle (diameter: 5.8 cm; 4.77°) was positioned in the centre of the screen and the objects occasionally bounced off its outer border. Bouncing against this inner orange circle induced a change in motion direction. Additionally, as soon as the *target objects* bumped against the inner orange circle, a colour change from yellow to blue (V, duration = 0.15 s), a sound (A, 440 Hz, duration = 0.15 s), or both would be elicited. In the control condition, the collision of the target bouncing against the inner circle did not elicit any sensory cue (NC). In Experiment 2, the technical setup changed, as we moved to a new lab. In this experiment, the stimuli were presented on a Dell E2414H monitor (width: 52.7 cm, height: 29.64 cm, 1,920 × 1,080 resolution, 60-Hz refresh rate) and the participant was positioned 119 cm away from the screen. Due to the change of the experimental setup, the size of the visual angles changed in Experiment 2: Blue and yellow smiley faces (diameter: 1 cm; ~0.48°) were moving within a grey circle (diameter: 20 cm, 9.6°). An orange circle (diameter: 5.8 cm; 2.79°) was positioned in the centre of the screen so that the objects occasionally bounced off its border. The objects moved at 2 pixels per frame (1.68° per s).

The movement of the objects followed Newtonian mechanics on a 2D plane. The object’s initial direction of movement was set randomly. The objects moved at 2 pixels per frame (3.6° per second). There was no friction between the objects; this prevented the objects from slowing down over time. However, the objects’ speed may vary in collisions. Despite this, there was a control implemented to keep the objects speed between 1 pixel per frame and 2.5 pixels per frame. This feature ensured that the targets would remain in motion during the experiment. Each target collided with the inner orange circle exactly once per trial. When a particular target collided with the inner circle, it moved toward that circle at a speed of 2 pixels per frame in a straight line. Such a motion sequence started every 2.55 s (i.e., target 1 started moving toward the inner circle at t = 2.55 s, target 2 at t =5.1 s, target 3 at t = 7.65 s, target 4 at t = 10.2 s, and target 5 at t = 12.75 s). As the duration of the motion toward the inner circle was random, the exact timing of the collisions was unpredictable for the observers.

#### Data analysis

Our experiment followed a 2 × 2 within-subject factorial design with the factors Visual Cue (present vs. absent), and Auditory Cue (present vs. absent). We chose this design as it allows for investigating the main effects of the tones as well as potential interactions between both factors. For the analysis, we calculated a 2 × 2 repeated-measures ANOVA including the factors *Visual Cue* and *Auditory Cue* run with the proportion of correctly identified targets (tracking accuracy) as dependent variables. Based on previous observations that visual cues improve tracking performance, there should be a main effect of the visual cues. Critically, if auditory cues also contribute to tracking performance, we should also observe a main effect of the auditory cue. Finally, a potential interaction would indicate that the cues do not contribute additively to tracking, which would require an additional inspection of the result pattern. A Greenhouse-Geisser correction was applied to the reported *p*-values. Post hoc *t*-tests were calculated in order to resolve interaction effects.

Outliers were computed for each condition separately and defined as 1.5 times the interquartile range away from the 75th or 25th percentile. Participants who had outlying data points (N = 3 in the audio-visual condition and N = 2 in the visual condition) were removed from the analysis.

### Results

**Fig. 2:**
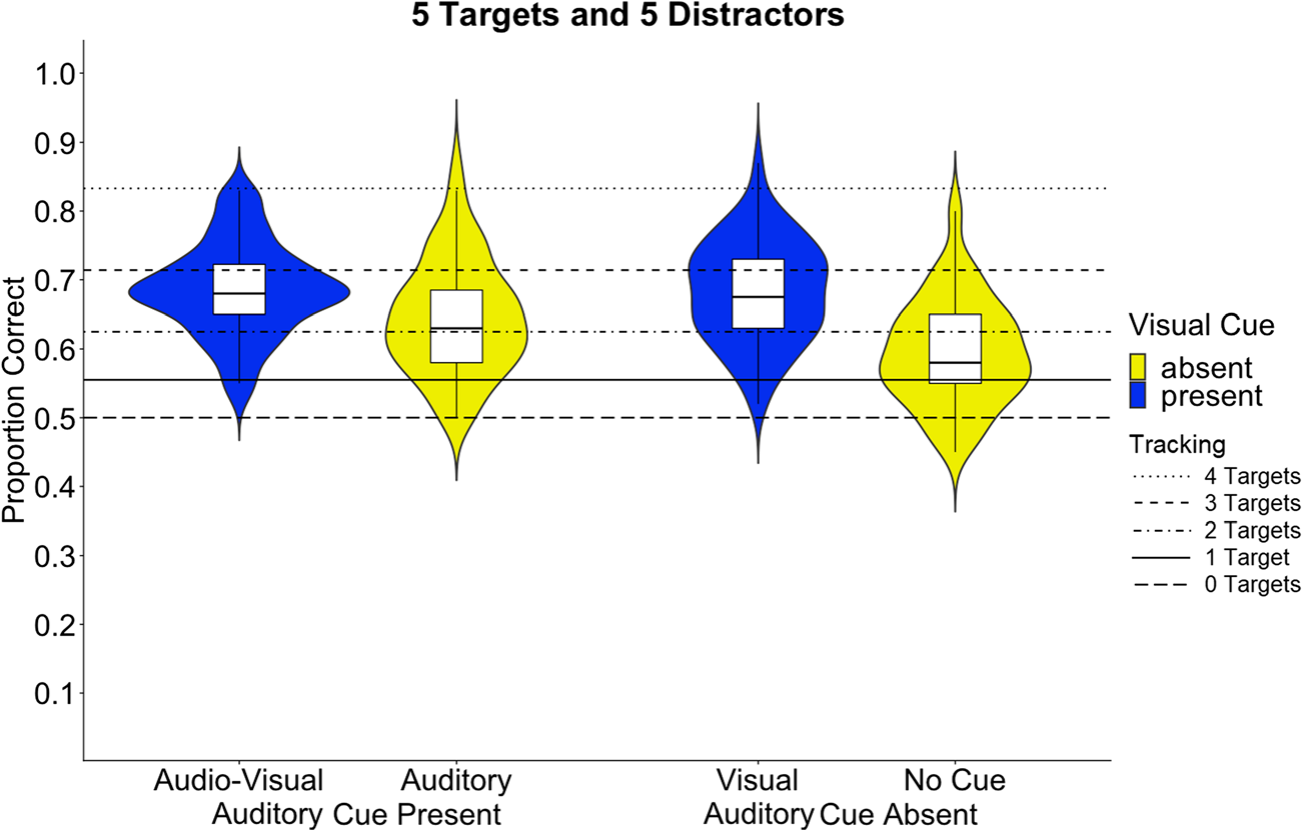
Mean proportion of correctly identified target objects during MOT for each condition: Audio-Visual cue, Auditory cue, Visual cue, and No cue. The blue colour indicates the presence of a visual cue, the yellow colour indicates the absence of a visual cue. The horizontal lines depict the tracking performance levels to be expected, according to Hulleman (2005), at tracking capacities of one, two, three, and four targets, with zero targets indicating chance level

The repeated-measures ANOVA including the factors *Visual Cue* (present, absent) and *Auditory Cue* (present, absent) revealed a significant two-way interaction between *Visual Cue* and *Auditory Cue, F*(1,67) = 13.33, *p* = .001; *η*_*p*_^*2*^ = .17 (see Fig. 2). This interaction indicates that visual cues, *M* = 0.68, *SE* = .01, as well as auditory cues, *M* = 0.65, *SE* = .01, improve tracking performance compared to the absence of any cues, *M* = 0.59, SE = .01; visual versus no-cue: *t*(67) = 9.11, *p* < .001; auditory versus no cue, *t*(67) = 5.39, *p* < .001. Furthermore, visual and audio-visual cues, *M* = 0.69, *SE* = .01, elicit more accurate tracking performance than auditory cues, visual versus auditory: *t*(67) = 4.22, *p* < .001; audio-visual versus auditory: *t*(67) = 4.11, *p* < .001. However, the audio-visual cue did not elicit any gain in tracking accuracy compared to the visual cue, *t*(67) = .26, *p*

Further, the main effects of *Visual Cue*, *F*(1,67) = 82.23, *p* < .001; *η*_*p*_^*2*^ = .55, and the main effect of *Auditory Cue* were significant, *F*(1,67) = 23.15, *p* < .001, *η*_*p*_^*2*^ = .26. Tracking performance was higher when visual cues were present (*M* = .68, *SE* = .008) as well as when auditory cues were present (*M* = .66, *SE* = .008) compared to when they were absent (visual *M* = .62, *SE* = .008; auditory *M* = .63, *SE* = .008).

### Discussion

The results of Experiment 1 suggest that auditory, visual, as well as audio-visual cues elicit more accurate tracking performance compared to when no cues were presented.

Crucially, we show that auditory cues can improve tracking performance compared to when no cues are delivered. This finding corresponds to the pip-and-pop effect observed in the visual search task (van der Burg et al., 2008) and might be explained by different theoretical accounts. The auditory cue might capture attention and turn the direction change of the target into an oddball among the multiple direction changes of the other moving objects. Consequently, these stimuli might be more salient for the participants, and therefore could be better distinguished from the presented distractors (Gao et al., 2021). Since the auditory cue was delivered every 2.5 s together with the collision of the target against the inner circle, the sound might induce a specific “rhythmicity” during the tracking phase that might have enhanced the predictability of the target (Barnhart et al., 2018).

The simultaneous presentation of the auditory cue and the visual direction change might act as a supramodal binding feature that allows the integration of the auditory and the visual modality and thus capture attention due to the increased saliency of the stimulus. Interestingly, a previous study has suggested that the ability to integrate an auditory cue with a visual event is limited to a singular pair of stimuli at a time (van der Burg et al., 2013); however, the temporal separation of the individual events in our experiment would allow such a mechanism.

Regarding the effectiveness of different cues, the finding of Experiment 1 suggests that audio-visual cues were not more effective than visual cues. Thus, it might be argued that the visual signal already provides the most reliable information about the target location so that auditory cues cannot add further effectiveness. However, previous studies have suggested that task-load might unfold multisensory integration, whereas other studies showed that multisensory integration was reduced under high load. Alsius et al. (2005) demonstrated that the McGurk effect is reduced under high-load conditions compared to the low-load condition. On the other hand, Santangelo and Spence (2007) have shown that a multisensory cue enhances the detection of a target at the cued location under both high and low perceptual load conditions. Strikingly, the multisensory cues remained effective under the high-load conditions whereas unimodal cues did not. Similarly, Lunn et al. (2019) have demonstrated that the detection of multisensory targets is enhanced compared to the unisensory targets under both high and low perceptual load. As these studies investigated search and rapid serial visual presentation (RSVP) paradigms that differ substantially from the MOT paradigm, a direct transfer of the result of Santangelo and Spence (2007) and Lunn et al. (2019) is not possible.

In Experiment 2, we therefore manipulated the tracking load that alters the attentional demands of the task (Meyerhoff et al., 2017).

The aim of this experiment is to further explore the effectiveness of different cues across different load conditions.

## Experiment 2

Experiment 2 was similar to Experiment 1, but we additionally manipulated the tracking load (i.e., the difficulty of the tracking task). To this end, we included a low-load (three target objects) and a high-load condition (five target objects) in our experimental design. If the effect of auditory and audio-visual cues on tracking is independent of the attentional load, we should replicate the result pattern of Experiment 1 for both tracking load conditions. In contrast, more pronounced effects of the cues would indicate a load dependency, as has been observed in related studies. This is in line with Alsius and co-authors (2005) who showed reduced Mc Gurk effects under high load compared to low load, as well as Santangelo and Spence (2007) who demonstrated that unisensory cueing effects disappear under high-load compared to low-load conditions.

### Participants

The final sample included in the data analysis were 28 participants (age range: 19–33 years, mean age: 21 years, *SD*: 2.52; 18 females). Data from two additional participants were removed due to outliers (see *Results* section). This sample size emerged from the following considerations. Based on the size of the effect of the auditory cue in Experiment 1 (*η*_*p*_^*2*^ = .26), we conducted a new power analysis (the correlations between measures was set to 0 as this relationship is already included in the effect size). This analysis suggests a sample size of N = 21. We over-recruited this number to compensate for potential exclusions. Eventually, we recruited 30 students as participants for Experiment 2. All participants had normal to corrected vision. Informed consent was obtained from all participants. The participants received course credits for participation.

### Apparatus, stimuli, procedure and experimental design

Apparatus, stimuli and procedure were identical to Experiment 1 with the following exceptions. We manipulated the number of to-be-tracked objects. The participants tracked three or five target objects among seven or five distractors, respectively (i.e., there were always ten moving objects in the display).

The experiment consisted of 16 blocks, which were divided in the no-cue, the auditory cue, the visual cue, and the audio-visual condition, separately for high and low load (2 × 4 = 8 conditions). Each block consisted of one of these conditions and was repeated twice, thus resulting in 16 blocks. Participants were trained on each experimental block prior to the main experiment. Therefore, three training trials were presented prior to ten main experimental trials in order to make participants familiar with the task in the first half of the experiment (blocks 1–8). Counterbalanced across participants, these blocks were presented in four different orders by presenting low-load and high-load blocks successively in each condition: (1) AV, V, NC, A; (2) NC, A, V, AV; (3) V, AV, A, NC; and (4) A, NC, AV, V.

In total, the participants completed 24 training trials, and 160 experimental trials, consisting of 20 experimental trials for each sensory and load condition. One block lasted for approximately 3 min and 25 s.

### Data analysis

The average proportion of correct MOT scores were calculated separately for each cue and load condition. We conducted a 2 × 2 × 2 repeated-measures ANOVA with the independent variables visual cue (present, absent), auditory cue (present, absent), and load (three targets, five targets). A Greenhouse-Geisser correction was applied to the reported *p*-values. Post hoc t-tests were calculated in order to resolve interaction effects. Identically to Experiment 1, outliers were computed for each condition separately and defined as MOT values 1.5 times the interquartile range away from the 75th or 25th percentile. Participants who had outlying data points were removed from the analysis (N = 2).

#### Results

**Fig. 3:**
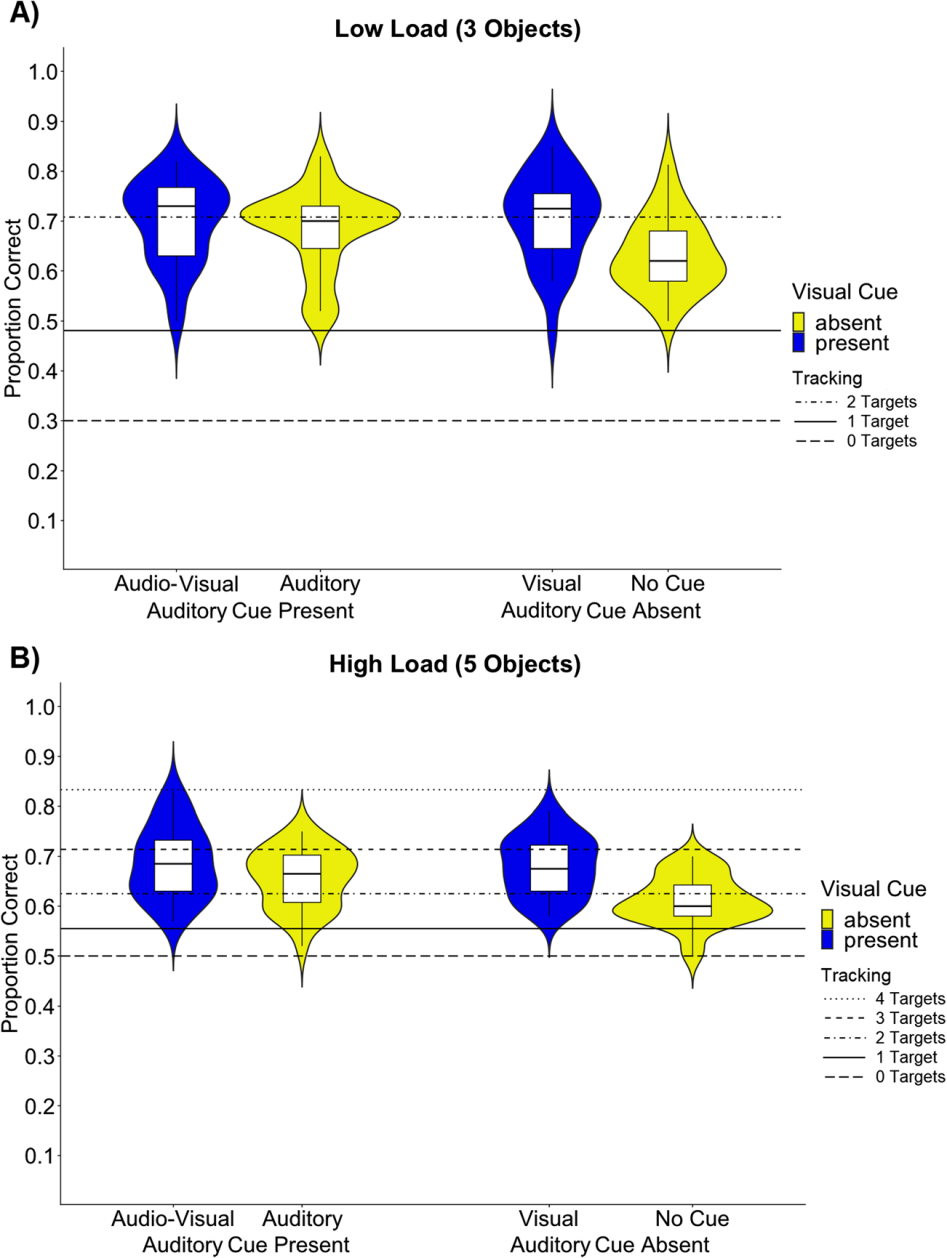
Mean proportion of correctly identified target objects for each condition (cue and load) in the low-load condition (**A**) and in the high-load condition (**B**): Audio-Visual cue, Auditory cue, Visual cue, and No cue. The blue colour indicates the presence of a visual cue, the yellow colour indicates the absence of a visual cue. The horizontal lines depict the tracking performance levels to be expected, according to Hulleman (2005), at tracking capacities of one, two, three, and four targets, with zero targets indicating chance level

The three-way interaction was not significant, *F*(1,27) = .27, *p* = .608, *η*_*p*_^*2*^ = .01, suggesting that the load manipulation does not modulate the effect of auditory and visual cues. Importantly, however, replicating Experiment 1, there was a significant interaction between the factors *Visual cue* and *Auditory cue*, *F*(1,27) = 10.55, *p* = .003, *η*_*p*_^*2*^ = .28. Matching the results pattern of Experiment 1, this interaction indicated that both visual and auditory cues improved tracking performance, but that there was no benefit emerging from audio-visual cues beyond the level of visual cues; visual cues versus no cues, *t*(27) = 7.21, *p* < .001; auditory cues versus no cues, *t*(27) = 4.25, *p* < .001; visual cues versus auditory cues, *t*(27) = 2.05, *p* = .050; audio-visual cues versus auditory cues: *t*(27) = 2.09, *p* = .046; audio-visual cues versus visual cues: *t*(27) = .17, *p* = .868; audio-visual cues versus no cue: *t*(27) = 6.06, *p* <.001 (see Fig. 3a, b).

As expected, the main effect of *Load* was significant as well, *F(*1,27) = 6.47, *p* = .017, *η*_*p*_^*2*^ = .19, which confirms that the manipulation of the tracking load successfully altered tracking difficulty. Overall, the proportion of accurately tracked objects was higher in the low-load conditions than in the high-load conditions; Low load: *M* = 0.68, *SE* = .012, High Load: *M* = 0.66, *SE* = 0.01. Moreover, the main effect of the *Visual cue*, *F*(1,27) = 32.31, *p* < .001, *η*_*p*_^*2*^ = .55, and the main effect of the *Auditory cue* were significant, *F*(1,27) = 9.24, *p* = .005, *η*_*p*_^*2*^ = .26. Participants’ performance was higher when visual cues were present (*M* = .69 , *SE* = .01), as well as when auditory cues were present (*M* = .68, *SE* = .01), compared to when they were absent (visual *M* = .64, *SE* = .01; auditory *M* = .65, *SE* = .01).

### Discussion

The results of Experiment 2 replicate the interaction effect observed in Experiment 1: Participants tracked a higher number of objects when auditory, visual or audio-visual cues were applied during tracking. Similar to Experiment 1, the results showed that participants were better able to track objects with visual cues than auditory cues. Moreover, visual and audio-visual cues did not elicit different tracking performances. Nevertheless, auditory cues elicited better performance compared to the no-cue condition (baseline). A main effect of *Load* suggested a higher proportion of correctly tracked objects with three target objects than five target objects. The absence of any interaction involving the load factor further suggests that this load effect is equally pronounced across all cue conditions. Thus, our findings suggest that load does not modulate the impact of sensory cues in MOT.

## General discussion

In two experiments, we aimed to understand whether auditory and/or audio-visual cues that coincide with visual direction changes during tracking improve MOT performance. As previous research has already demonstrated the effectiveness of visual cues in maintaining targets (Bae & Flombaum, 2012), we also included purely visual cues in our study in order to compare the effectiveness of the different cues. To investigate this question, we presented auditory, visual and audio-visual cues when a target was bouncing against the inner circle during tracking and compared those conditions to a baseline condition in which no cues were presented. In Experiment 1, the participants were asked to track five target objects among five distractor objects, whereas we manipulated tracking *Load* by asking participants to track either five or three objects (out of ten objects) in Experiment 2. The *Load* factor has been included in order to investigate whether the cues might be more efficient under a specific load condition. Previous research has shown that multisensory speech illusions, such as the McGurk effect, diminish under high-load conditions (Alsius et al., 2005), whereas spatial cueing experiments demonstrate reliable validity effects under both low- and high-load conditions when audio-visual cues are presented (Santangelo & Spence, 2007). As the tracking demands under high-load conditions could be so high that they even interfere with tasks such as scene classification (Cohen et al., 2011), it thus might be possible that sensory cues do not realise their full potential under such conditions. Contrary to such potential modulations of the cue effectiveness, however, we observed that auditory, visual or audio-visual cues were equally effective across both set sizes. Further, consistently across both experiments, visual cues were more effective than auditory cues, and combining both cues did not improve tracking performance beyond the level of the purely visual cues. The observation that auditory cues improved tracking performance in both high- and low-load conditions compared to the no-cue condition shows that non-visual cues are able to guide attention under different load conditions in a tracking environment. This contrasts with other studies that demonstrated that such cues did not alter task performance under high-load conditions (Santangelo & Spence, 2007). Various reasons might account for the differing impact of auditory cues, such as the different experimental scenarios, the salience of the stimuli, the presented paradigm, and the tasks. For instance, in our experimental design the auditory cue was presented temporally aligned with the visual target, which might have elicited an enhancement of the visual target event by integrating both auditory and visual events. Furthermore, in our paradigm, the target objects bounced in a *regular time interval* against the inner circle, and thus rhythm, also enhanced via the temporally coincident cues, might offer an additional signal that allowed the generation of further predictions about the location of the target object.

According to the predictive coding model, internal models are constantly updated based on the prediction of incoming sensory input. The conclusion of a causal structure in the task, for example, the bouncing effect of the target at regular time intervals, “allows grouping (or segregating) sensory inputs from different modalities according to their common (or different) causal origin. Solving this causal inference problem would result in the formation of multisensory perceptual representations” (Soto-Faraco et al., 2019, p. 21; Noppeney, 2021 for a review). Underlying neural mechanisms of multisensory integration have been recorded in different cortical areas along the cortical hierarchy (see Noppeney, 2021, for a review), including early sensory areas as well as higher cortical regions (Molholm et al., 2002; Murray et al., 2016).

While our experiments demonstrate that observers in principle can benefit from visual and/or auditory cues that allow a re-identification of targets, they were not designed to disentangle whether bottom-up and top-down attentional processes (or a combination of both) improved the tracking performance. This is because we informed our participants prior to the experiment that only targets that bounce against the inner circle would be accompanied by a sensory cue, whereas distractors would never be paired with a sensory cue information. Nevertheless, our finding that auditory cues that coincide with the direction changes of the targets improves tracking mimics previous results of auditory cues facilitating visual search rates (e.g., van der Burg et al., 2008). Crucially, a previous study has demonstrated that the ability to integrate an auditory cue with a visual event is limited, suggesting that one visual stimulus can be associated with a visual target at a time (van der Burg et al., 2013). Indeed, we demonstrate that the bouncing of one target at a time elicits an auditory cue and improves tracking performance. Regarding the attentional processes, there is evidence that the coinciding tones in the visual search experiments automatically captured attention (i.e., bottom-up). For instance, Matusz and Eimer (2011) studied an audio-visual adaptation of the spatial cueing paradigm (Folk et al., 1992). In this task, a colour change of a spatial cue that matched the colour of the target or a colour change of a spatial cue that did not match any colour of the visual search objects was accompanied by a tone. Following this cue, the participants were asked to visually search for a coloured target. Matusz and Eimer (2011) showed that the spatial cueing effect (shorter response latencies for matching than mismatching cue and target locations) was more pronounced in tone-present than tone-absent trials and occurred irrespective of whether the cue corresponds to the colour of the target or did not match any visual search objects. As the cueing effect emerged independently of task requirements (searching for a coloured bar vs. searching for a specific colour), the processing of the cues can be considered largely bottom-up. In order to disentangle bottom-up and top-down attention in our paradigm, future research should attempt to also investigate the impact of auditory cues when they coincide with the direction changes of distractors rather than targets. If tones that coincide with the distractors have a detrimental effect on tracking, such a finding would suggest automatic guidance.

One interesting observation in our results is that the audio-visual cues did not elicit more accurate tracking performance than purely visual cues, although both auditory and visual cues improve performance compared to the baseline without any cues. This contrasts with studies that document enhanced performance of multisensory cues under low and even high perceptual load conditions and reduced effectiveness of unisensory cues under high perceptual load (Santangelo & Spence, 2007).

Several factors could contribute to this lack of an enhanced audio-visual cueing effect: First, in order to increase the probability of audio-visual cues being relevant during object tracking, it might be important to follow the principle of “inverse effectiveness” (Stein & Stanford, 2008, p. 257). According to this principle, highly salient individual cues will be easily detected and localised. Thus, their combination has a proportionately moderate effect on neural-behavioural mechanisms. By contrast, weak cues evoke comparatively few neural impulses, and their responses are therefore subject to fundamental enhancement when stimuli are combined (Stein & Stanford, 2008). In these cases, the multisensory response can exceed the arithmetic sum of their individual responses and can have a significant positive effect on behavioural performance by increasing the speed and likelihood of detecting and locating an event. In order to test the principle of inverse effectiveness and apply it to our paradigm, it might be argued that the combination of sensory cues would be more effective, if the individual cues were of reduced salience. Therefore, follow-up studies that vary the relative weight of the visual cues appear to be a promising avenue to further investigate the effectiveness of auditory and audio-visual cues during MOT.

Second, it might be argued that task load modulates the integration of sensory cues; however, this appears not to be likely when considering our data. When we compared our low-load (three targets) and high-load (five targets) conditions in Experiment 2, there was no interaction between load and the sensory cues. This suggests that the relative effectiveness of the audio-visual cues was not modulated by the load. Nevertheless, future studies could include a higher number of target objects in the experimental design as well as a higher overall number of objects (i.e., increasing the display density; see Bettencourt & Somers, 2009). For the moment, however, our finding is in line with previous studies in which attentional load did not modulate the effectiveness of multisensory cue information. For instance, Santangelo and Spence (2007) observed that spatial cueing effects were elicited by multisensory cues, irrespective of the perceptual load condition. Combining the results across paradigms, it therefore might be argued that the effect of sensory cues is “immune” against task-load conditions.

A further candidate for extending our current study would be to investigate the guidance of attention by auditory and audio-visual cues within the multiple identity paradigm (MIT; Horowitz et al., 2007; Oksama & Hyönä, 2004, 2008). In this paradigm, each object has an individual identity matching real-world scenarios more closely than indistinguishable objects (Oksama & Hyönä, 2016). A recent model explaining such a tracking of identities (Model Of Multiple Identity Tracking (MOMIT); Li et al., 2019) argues in favour of a cooperative use of attention, eye movements, perception, and working memory for dynamic tracking. Tracking appears more serial when high-resolution information needs to be sampled and maintained for discriminating the targets, whereas it appears more parallel when low-resolution information is sufficient. Combining the theoretical ideas of MOMIT with the multisensory approach of our work might allow the identification of which processes that contribute to tracking are affected by the auditory or audio-visual cues.

To conclude, our findings suggest that visual and auditory cues are able to enhance tracking performance. However, we did not find any evidence for multisensory cues enhancing performance compared to unisensory cues in a MOT task. Further experiments are necessary in order to understand the integration principles of multisensory cues in MOT as well as the bottom-up versus top-down nature of their impact.
